# The Behaviour of the Pelvic Articulations in the Mechanism of Parturition

**Published:** 1855-01

**Authors:** Matthews Duncan


*The Behaviour of the Pelvic Articulations in the Mechanism of Parturition.* By Dr. Matthews Duncan.—In the guinea pig, there takes place at the time of parturition a very considerable separation of the pubic bones, the ligamentous tissue of the symphysis stretching in this small animal to the extent of an inch, or even more. In the guinea pig, the motions of the iliac bones are analogous to those of abduction of the limbs. In the cow, such movements are absent. The symphysis pubis is consolidated by bony union, and consequently abduction of the iliac bones is impossible. But the sacroiliac joints become much relaxed, as also the sacrosciatic ligaments, and by these changes the ilia become extensively moveable upon the sacrum in an antero-posterior direction, the motions being analogous to those of flexion and extension in the limbs. The final result of these changes in the guinea pig and the cow is to enlarge the genital passage for the transmission of the calf; and these two animals present, respectively, characteristic examples of the two different sets of changes by which this result is obtained in different animals.

In the latter half of pregnancy in the human female, the soft tissues contributing to form the pelvic joints are found softened, as if by serous infiltration; and the joints are consequently relaxed. The softening of these tissues is accompanied by their increase of thickness, a change which will itself have, as a necessary consequence, the separation of the bones and enlargement of the pelvic circle. And this favorable circumstance, along with others connected with the motions of the joints, forms an important part of the explanation of some cases of delivery, by a simpler operative procedure than might at first have been considered necessary. Sometimes this thickening is to a very great extent, as in the cases of Madame Boivin and others, where the pubic bones were separated an inch or more.

The movements in the pelvic joints in the human female are the same as those occurring in man as well, only in a minor degree, as has been demonstrated by Mr. Zaglas. They may be described as consisting in the elevation and depression of the symphysis pubis, the ilia moving upon the sacrum. By the elevation of the symphysis (or projection forwards of the promontory of the sacrum), the angle of inclination of the pelvis is diminished, and the conjugate diameter of the brim is lessened to the extent of one, or even two lines; the corresponding diameter of the outlet is increased about twice as much. This different ratio of the effects of the motion upon the brim and outlet, results from the fact of the centre of motion being much nearer the promontory than the apex of the bone (in the second sacral vertebra). The promontory, therefore, will describe in its motions an arc of a smaller radius than the apex.

That the alteration of the dimensions of the brim and outlet by these movements is not insignificant, but the reverse, is a proposition which every obstetrician will confirm It only remains, then, to be observed, how these alterations correspond with the phenomena of the progress of the child in parturition. Now, in the erect position, the brim of the pelvis is in its enlarged condition, the symphysis pubis being then depressed, while the outlet is correspondingly contracted. In the course of the first stage of labor, while the head is pressing into the brim, the human female is generally standing, sitting, or lying on her back, or in an easy position. But as soon as the head has descended into the pelvis and infringed upon the sensitive vagina, then forcing efforts accompany the pains. These forcing efforts consist, in great part, of powerful contractions of the anterior abdominal muscles, the effect of which, especially the action of the two recti-muscles, will be to tilt up the symphysis pubis, thus throwing the promontory forwards, contracting the brim, and enlarging the outlet, and diminishing the angle of inclination of the pelvis. To all these changes, the position usually assumed by the female in the second stage of labor will contribute. For the simple bending of the body forwards (even in man), has for its effect the tilting upwards of the apex of the sacrum, and enlargement of the outlet. And, it is a curious fact, that a woman in her forcing pains, in the second stage, is found to draw up her legs, and bend her body forwards, thus inducing changes in her pelvis, which facilitate the advance of the child in that stage.

These motions of the pelvic bones in the human female agree with those taking place in the cow at the time of labor. The changes occurring at this time in the guinea pig, find their analogies in the altered condition of the symphysis in woman—changes which in her are generally only to a small extent.—*Edinburgh Quarterly, from Dublin Quarterly Journal of Medical Science, August,* 1854.

